# CT-Based Peritumoral and Intratumoral Radiomics as Pretreatment Predictors of Atypical Responses to Immune Checkpoint Inhibitor Across Tumor Types: A Preliminary Multicenter Study

**DOI:** 10.3389/fonc.2021.729371

**Published:** 2021-10-18

**Authors:** Shuai He, Yuqing Feng, Qi Lin, Lihua Wang, Lijun Wei, Jing Tong, Yuwei Zhang, Ying Liu, Zhaoxiang Ye, Yan Guo, Tao Yu, Yahong Luo

**Affiliations:** ^1^ Department of Medical Imaging, Cancer Hospital of China Medical University, Liaoning Cancer Hospital and Institute, Shenyang, China; ^2^ Department of Oncology, The Fifth People’s Hospital of Shenyang, Shenyang, China; ^3^ Department of Radiology, First Affiliated Hospital of Xiamen University, Xiamen, China; ^4^ Department of Radiology, General Hospital of Northern Theater Command, Shenyang, China; ^5^ Department of Radiology, Tianjin Medical University Cancer Institute and Hospital, Tianjin, China; ^6^ Prognostic Diagnosis, GE Healthcare China, Beijing, China

**Keywords:** radiomics, CT, peritumoral, immune checkpoint inhibitor, atypical responses

## Abstract

**Objective:**

To develop and validate a new strategy based on radiomics features extracted from intra- and peritumoral regions on CT images for the prediction of atypical responses to the immune checkpoint inhibitor (ICI) in cancer patients.

**Methods:**

In total, 135 patients derived from five hospitals with pathologically confirmed malignancies receiving ICI were included in this retrospective study. Atypical responses including pseudoprogression (PsP) and hyperprogression disease (HPD) were identified as their definitions. A subgroup of standard progression disease (sPD) in 2018 was also involved in this study. Based on pretreatment CT imaging, a total of 107 features were extracted from intra- and peri-tumoral regions, respectively. The least absolute shrinkage and selection operator (Lasso) algorithm was used for feature selection, and multivariate logistic analysis was used to develop radiomics signature (RS). Finally, a total of nine RSs, derived from intra-tumoral, peri-tumoral, and combination of both regions, were built respectively to distinguish PsP *vs*. HPD, PsP *vs*. sPD, and HPD *vs*. sPD. The performance of the RSs was evaluated with discrimination, calibration, and clinical usefulness.

**Results:**

No significant difference was found when compared in terms of clinical characteristics of PsP, HPD, and sPD. RS based on combined regions outperformed those from either intra-tumoral or peri-tumoral alone, yielding an AUC (accuracy) of 0.834 (0.827) for PsP *vs*. HPD, 0.923 (0.868) for PsP *vs*. sPD, and 0.959 (0.894) for HPD *vs*. sPD in the training datasets, and 0.835 (0.794) for PsP *vs*. HPD, 0.919 (0.867) for PsP *vs*. sPD, and 0.933 (0.842) for HPD *vs*. sPD in the testing datasets. The combined RS showed good fitness (Hosmer–Lemeshow test p > 0.05) and provided more net benefit than the treat-none or treat-all scheme by decision curve analysis in both training and testing datasets.

**Conclusion:**

Pretreatment radiomics are helpful to predict atypical responses to ICI across tumor types. The combined RS outperformed those from either intra- or peri-tumoral alone which may provide a more comprehensive characterization of atypical responses to ICI.

## Introduction

The novel development of the immune checkpoint inhibitor (ICI) is now approved in a variety of solid tumors, including melanoma, non-small cell lung cancer (NSCLC), and urothelial and microsatellite instability-high (MSI) cancer, represented by programmed cell death-1 (PD-1) and programmed cell death ligand-1 (PD-L1), which became a crucial therapeutic option to improve prognosis (1). Unlike chemotherapy and tyrosine kinase inhibitor (TKI), ICI plays an antitumor role by blocking the immune checkpoint and enhancing the activity of autologous T cells (2). These effects occur through the restart of intrinsic immune actions, and the efficacy of these effects is strictly associated with the appearance of hypoxia, necrosis, and inflammation at the tumor sites (3). Meanwhile, these biological processes can affect the immune system and adjust antitumor responses, giving rise to atypical responses, including pseudoprogression (PsP) and hyperprogression disease (HPD) (4–7).

During tumor assessment, PsP occurs as a shrinkage in tumor burden after increasing in size or the presence of new lesions (8, 9), whereas HPD is presented as an acceleration of tumor growth after the initiation of immunotherapy, as compared to the period before treatment initiation used as a reference (10–12). Most similarly, all PsP, HPD, and standard progression disease (sPD) patients share common imaging with tumor enlargement at initial radiography assessment. Dissimilarly, compared with sPD and HPD, PsP patients have good clinical outcomes with significant longer progression-free survivals and overall survivals (13, 14). Thus, distinguishing PsP from sPD or even HPD will extremely help evaluate the efficacy of ICI and avoid either premature withdrawal of the treatment or prolonging ineffective treatment. Unfortunately, the identification of a reliable predictive biomarker of atypical responses to immunotherapy across various solid tumors remains an unmet need in clinic practice so far.

Routine standard-of-care CT scans are a noninvasive clinical examination tool for tumor diagnosis, staging, and monitoring treatment response. CT imaging-based radiomics can characterize both intra-tumoral and peri-tumoral heterogeneity from digital images to build mathematical formulas that reflect the underlying pathophysiology (15). Nowadays, radiomics has been successfully applied to the prediction of tumor histology, risk of lymph node metastasis, genetic mutation subtypes, and decoding of PD-L1 expression in cancer patients (16–19). However, investigations using radiomics on prediction of atypical responses to cancer immunotherapy are rather rare. Recently, Wang et al. used CT radiomics to identify five PsP cases from 50 melanoma patients treated with anti-cytotoxic T-lymphocyte antigen-4 (CTLA-4) inhibitor (20). Nonetheless, it needs a larger sample to confirm the ability of radiomics to predict PsP before immunotherapy.

In this study, we aim to evaluate the predictive value of radiomics, including intra- and peri-tumoral features that distinguish among PsP, HPD, and sPD patients, which may assist clinicians in the precision management of personalized immunotherapy.

## Material and Methods

### Patients

This retrospective multicenter study was approved by each participating institutional review board, and the prerequisite for obtaining informed consent was waived. Data were collected from February 2017 to April 2020 in patients with pathologically proven malignant solid tumors who had been treated with ICI, alone or in combination with chemotherapy.

Four hundred and sixty-three consecutive patients from five Chinese Hospitals were identified. The inclusion criteria were as follows: (1) CT scan prior to the initiation of ICI in less than 2 weeks; (2) at least two cycles of ICI; and (3) all solid tumors were pathologically confirmed. The exclusion criteria were as follows: (1) with history of any other concurrent malignancies; (2) no measurable lesion or with obvious artifacts on CT images; and (3) without a previous and/or follow-up CT scan.

### Definitions of Pseudoprogression and Hyperprogression

**Figure 1:**
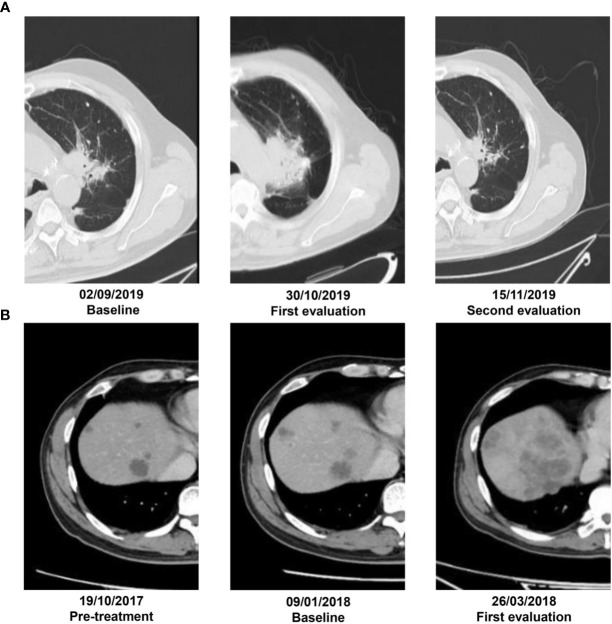
**(A)** A patient with adenocarcinoma by puncture biopsy pathology who was receiving nivolumab therapy. Irregular lesion in the left upper lobe with a diameter of 3.2 cm on the baseline CT scans. By 6 weeks of anti-PD-1 therapy, the lesion increased in diameter of 5.4 cm on the first CT evaluation. At 8 weeks of therapy, it had decreased in size by 2.2 cm. **(B)** Another 64-year-old male was treated with pembrolizumab on January 29, 2018, for liver metastasis from colorectal cancer. It had SD prior to the initiation of immunotherapy but developed rapid tumor growth with appearance of new lesions on the first follow-up and experienced more than two-fold increase from pretreatment tumor growth *versus* treatment.

Immune Response Evaluation Criteria in Solid Tumors (iRECIST) were used to evaluate the response of tumors (21). In our datasets of patients, PsP **(** **Figure 1A** **)** was defined as immune unconfirmed progressive disease (iUPD) during evaluation and further response classified as immune complete response (iCR), immune partial response (iPR), or immune stable disease (iSD) (22). HPD **(** **Figure 1B** **)** criteria, which are as follows in accordance with previous studies: 1) progression at first post-ICI, 2) increase in tumor size over 50%, and 3) over two-fold increase in progression rate (23, 24). We also defined other immune confirmed progressive diseases (iCPDs) except for HPD as sPD by iRECIST criteria. All measurable target lesions (≥10 mm in the longest diameter for non-nodal lesions and ≥15 mm in the short axis for nodal lesions) allow up to two lesions per organ, and five lesions in total as in iRECIST criteria were used for analysis.

**Figure 2:**
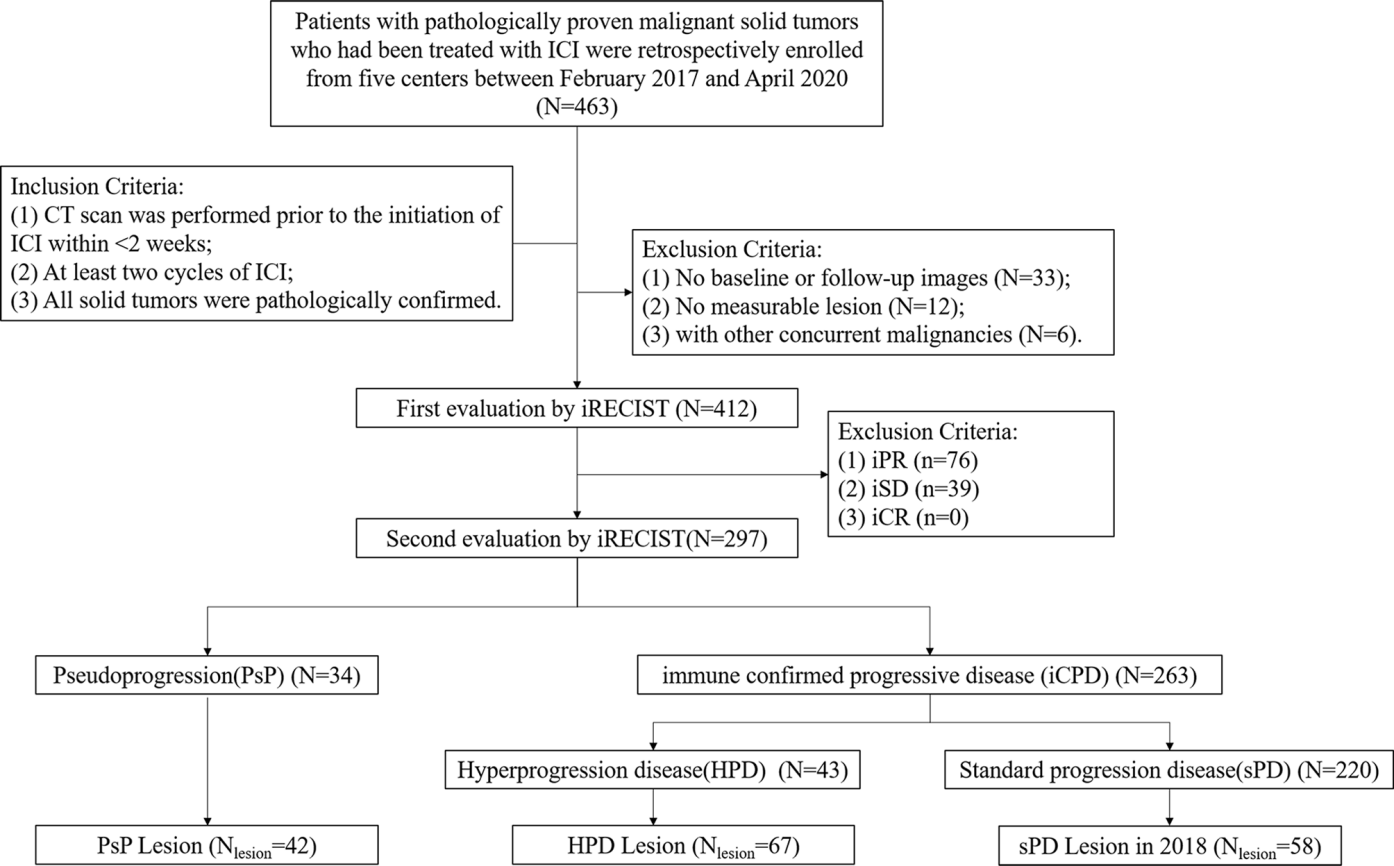
Flowchart of the patient selection procedure.

According to the definitions, 34 patients with 42 target lesions undergo PsP, and 43 patients with 67 target lesions experience HPD. A subgroup of sPD (58 out of 220) in 2018 was also included in this study. Finally, the training datasets were recruited from the Liaoning Cancer Hospital while the testing datasets involved patients from The Fifth People’s Hospital of Shenyang, First Affiliated Hospital of Xiamen University, General Hospital of Northern Theater Command, and Tianjin Cancer Hospital. The flowchart of patient selection procedure is showed in **Figure 2**.

#### CT Acquisition

The pretreatment CT scans were acquired on varied datasets of CT scanners **(Supplemental Data)**.

### Segmentation and Feature Extraction

Before segmentation, all images were resampled to a common voxel spacing of 1 mm × 1 mm × 1 mm by using the linear interpolation algorithm to construct new data points within the range of discrete datasets of known data points to standardize spacing across all images (25).

Then, the region of interest (ROI) was delineated manually along the tumor contour slice by slice on pretreatment CT images by reader 1 (HS with 9 years of experience) who were blinded to diagnosis and clinical information, using an open-source software (ITK-SNAP, version 3.6.0, http://www.itksnap.org/). The morphologic operation of dilation was then performed to capture the information outside the lesion up to a radial distance of 5 mm; normal tissue or surrounding organs were subsequently excluded from the contours.

Subsequently, a total of 107 radiomics features, which regarded the image biomarker standardization initiative (IBSI) as reference (26), were extracted using A.K. software (Artificial Intelligence Kit, version 2.0.0, GE Healthcare, China) from each region, including the intra- and peri-tumoral ROI.

In order to investigate the reproducibility of the radiomics features obtained by different readers, different times, and different tumor regions (intratumoral and peritumoral), intra- and interclass correlation coefficients (ICCs) were used to assess the reproducibility of the radiomics features extracted from 30 randomly chosen patients. To assess the inter-reader reproducibility, the ROI delineation was performed by two oncologic radiologists (reader 1 and reader 2, HS with 9 years and WLH with 7 years of experience), respectively. To evaluate the intra-reader reproducibility, reader 1 repeated the ROI delineation at a 1-month interval.

### Radiomics Signature Building

To reduce overfitting or selection bias, we adopted a series of methods for dimensionality reduction and feature selection before modeling. In the first step, features of ICCs >0.75 for both inter-reader and intra-reader that were considered a relatively high inter-reader and intra-reader variability in the segmented ROI, were included in subsequent analysis. Subsequently, Spearman correlation analysis was conducted to remove the redundant features which were highly correlated(|r|>0.90) with other features. Then, the least absolute shrinkage and selection operator (LASSO) regression algorithm with penalty parameter tuning was applied with 10-fold cross validation to select the most useful predictive features with a non-zero coefficient.

**Figure 3:**
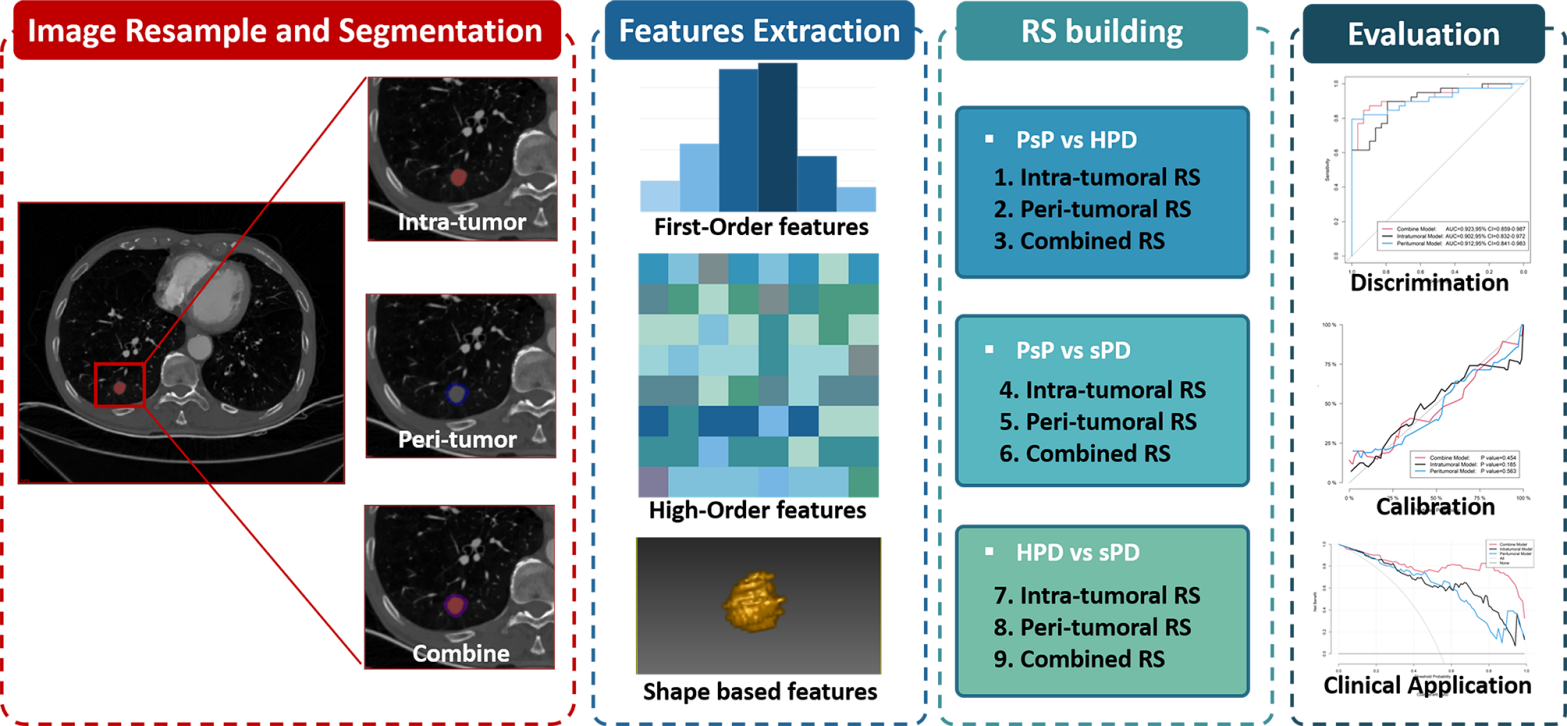
The radiomics workflow including tumor segmentation, feature extraction, radiomics signatures construction, and performance evaluation.

Then, a total of nine radiomics signatures (RSs), including intra-tumoral RS, peri-tumoral RS, and combined (intra-plus-peri-tumoral) RS for distinguishing PsP *vs*. HPD, PsP *vs*. sPD, and HPD *vs*. sPD, were built respectively *via* multivariate logistic analysis using the selected optimal feature sets in the training datasets and then tested in the testing datasets. The workflow of the radiomics analysis is shown in **Figure 3**.

### Performance Evaluation

The performance of the RSs was evaluated with discrimination, calibration, and clinical application in both training and testing datasets.

#### Discrimination Degree

Receiver operating characteristic (ROC) curves were plotted, and the area under the ROC curve (AUC) with a 95% confidence interval (CI) was used to assess the diagnostic performance in discriminating PsP *vs*. HPD, PsP *vs*. sPD, and HPD *vs*. sPD in both training and testing datasets. The optimal cutoff of the RSs calculated from the training datasets based on the maximum Youden’s index was then applied in the testing datasets. The accuracy (ACC), sensitivity (SEN), specificity (SPE), positive predictive value (PPV), and negative predictive value (NPV) were calculated in both training and testing datasets.

#### Calibration Degree

Calibration curves were plotted in both training and testing datasets to explore the agreement between the observed outcome frequencies and predicted probabilities of the RSs. The Hosmer–Lemeshow test was used to determine the goodness of fit of the models, and p values of more than 0.05 were considered well-calibrated.

##### Clinical Application

Decision curve analysis (DCA) was conducted to assess the clinical usefulness by quantifying the net benefits at different threshold probabilities in both training and testing datasets.

### Statistical Analysis

All statistical analyses were performed using R language (version 3.5.1, https://www.r-project.org). Categorical variables between two or more groups were compared with the χ² test, and continuous variables between groups were compared with either Student’s t test or Mann–Whitney U test (for two groups, as appropriate) with Bonferroni correction (p < 0.017 indicated significance) or by ANOVA (for three groups). Categorical variables were presented as counts (percentage), and continuous variables were presented as mean (SD) or median (25%, 75%), as appropriate.

ICC was calculated using the “lme4” package. LASSO regression was performed using the “glmnet” package. Multivariate logistic regression was performed using the “rms” package. ROC curves were plotted using the “pROC” package. The calibration curve and Hosmer–Lemeshow test were conducted using the “ModelGood” package. Decision curve analysis was performed using the “dca. R” package.

## Results

### Patient Dataset

A total of 135 patients including PsP (N = 34), HPD (N = 43), and sPD (N = 58) were analyzed in this study. The most common tumor types included the respiratory system neoplasms (n = 73) and digestive system neoplasms (n = 31). In our population, the incidence rate of HPD (10.44%) is slightly higher than PsP (8.25%) in the whole datasets. More than half of the patients (n = 72) received ICI monotherapy, and 46.67% (n = 63) received combination chemo-immunotherapy.

There was no statistically significant difference in baseline age, gender, pre-chemotherapy or radiotherapy, monotherapy or plus chemotherapy, brain metastasis, bone metastasis, lung metastasis, or hepatic metastasis (all p > 0.05) among the three groups. The characteristics of 135 patients are summarized in **Table 1**.

**Table 1:** Baseline characteristics of 135 patients.

Characteristics	PsP (N = 34)	HPD (N = 43)	sPD (N = 58)	*p* value
**Age, median (range) years**	67 (52-81)	62 (45-77)	72 (57-87)	0.368
**Gender, No. (%)**				0.202
Male	29 (85.3)	32 (74.4)	39 (68.4)	
Female	5 (14.7)	11 (25.6)	18 (31.6)	
**Pre- chemotherapy or radiotherapy, No. (%)**				0.851
Yes	26 (76.5)	33 (23.3)	46 (80.7)	
No	8 (23.5)	10 (76.7)	11 (19.3)	
**Treatment strategy, No. (%)**				0.183
Monotherapy	19 (44.1)	17 (39.5)	21 (36.8)	
Combination therapy	15 (44.1)	26 (60.5)	36 (63.2)	
**Number of lines of prior systemic cancer therapy, No. (%)**				0.337
1	7 (20.6)	9 (20.9)	9 (15.8)	
≥2	27 (79.4)	34 (79.1)	48 (84.2)	
**Lung metastasis, No. (%)**				0.817
With	18 (52.9)	25 (58.1)	34 (59.6)	
Without	16 (47.1)	18 (41.9)	23 (40.4)	
**Brain metastasis, No. (%)**				0.124
With	2 (5.9)	7 (16.3)	3 (5.3)	
Without	32 (94.1)	36 (83.7)	54 (94.7)	
**Bone metastasis, No. (%)**				0.120
With	8 (23.5)	16 (37.2)	11 (19.3)	
Without	26 (76.5)	27 (62.8)	46 (80.7)	
**Liver metastasis, No. (%)**				0.240
With	5 (14.7)	18 (41.9)	18 (41.9)	
Without	29 (85.3)	29 (85.3)	25 (58.1)	

Psp, pseudoprogression; HPD, hyperprogression disease; sPD, standard progression disease.

### Development and Validation of Radiomics Signatures

#### RS for Discriminating PsP From HPD

After inter- and intra-reader reproducibility analysis, a total of 210/214 (104/107 from intra-tumoral and 106/107 from peri-tumoral) features showed stability with both intra- and inter-reader ICCs greater than 0.75; the details are shown in **Table 2** and **Supplementary Figure 1**.

**Table 2:** The process of features selection.

Radiomics signatures		Remained feature number			
		Extracted	ICC>0.75	Spearman (|r|<0.90)	LASSO (non-zero)
**PsP *VS.* HPD**	**Intra-tumoral**	107	104	45	4
	**Peri-tumoral**	107	106	36	4
	**Combined**	214	210	79	5
**PsP *VS.* sPD**	**Intra-tumoral**	107	106	41	7
	**Peri-tumoral**	107	106	42	8
	**Combined**	214	212	69	11
**HPD *VS.* sPD**	**Intra-tumoral**	107	104	47	7
	**Peri-tumoral**	107	106	43	9
	**Combined**	214	210	80	12

Psp, pseudoprogression; HPD, hyperprogression disease; sPD, standard progression disease; ICC, inter/intra-class correlation coefficient; LASSO, least absolute shrinkage and selection operator.

The most predictive and strongest features were remained after the process of feature selection (**Table S1**). Then, RSs were calculated for each patient *via* a linear combination of the selected features weighted by respective coefficients (the calculation formulas are shown in **Supplementary Formula**).

**Figure 4:**
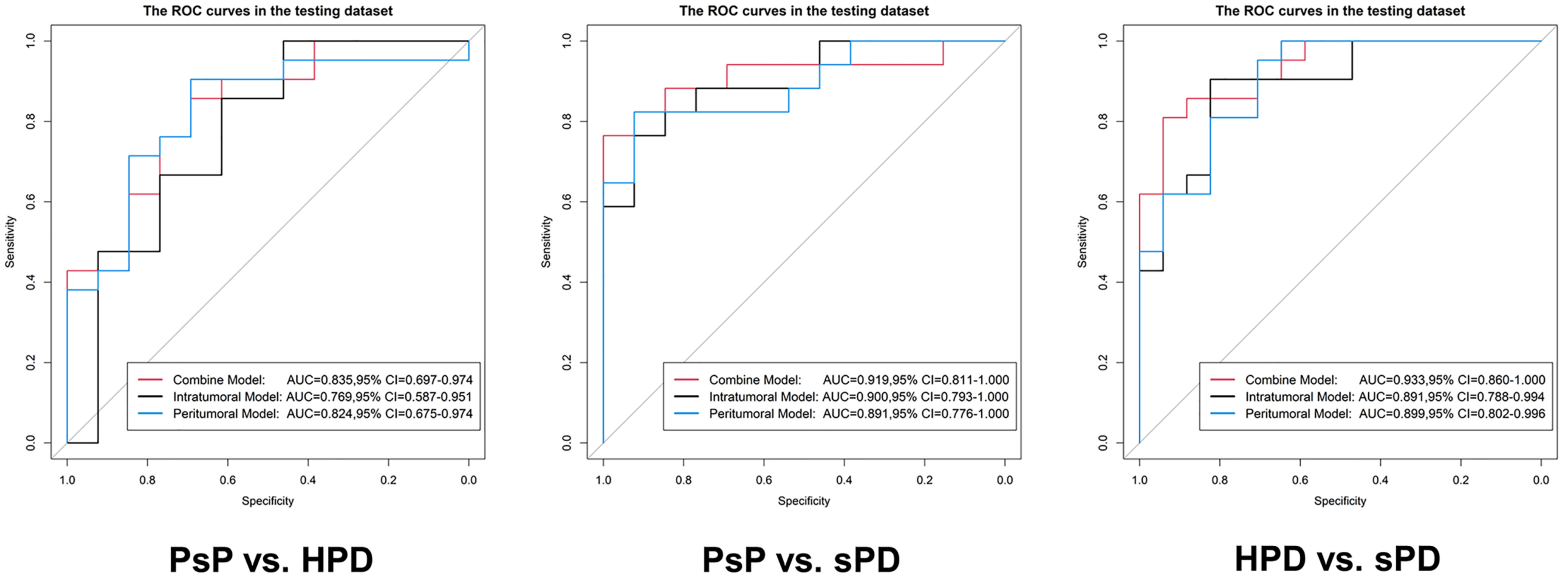
Evaluation of the predictive performance of the radiomics signatures in the testing datasets. In each ROC, the black curve is the ROC of the intratumoral model, the blue curve is the ROC of the peritumoral model, and the red curve is the ROC of the combine model.

**Figure 5:**
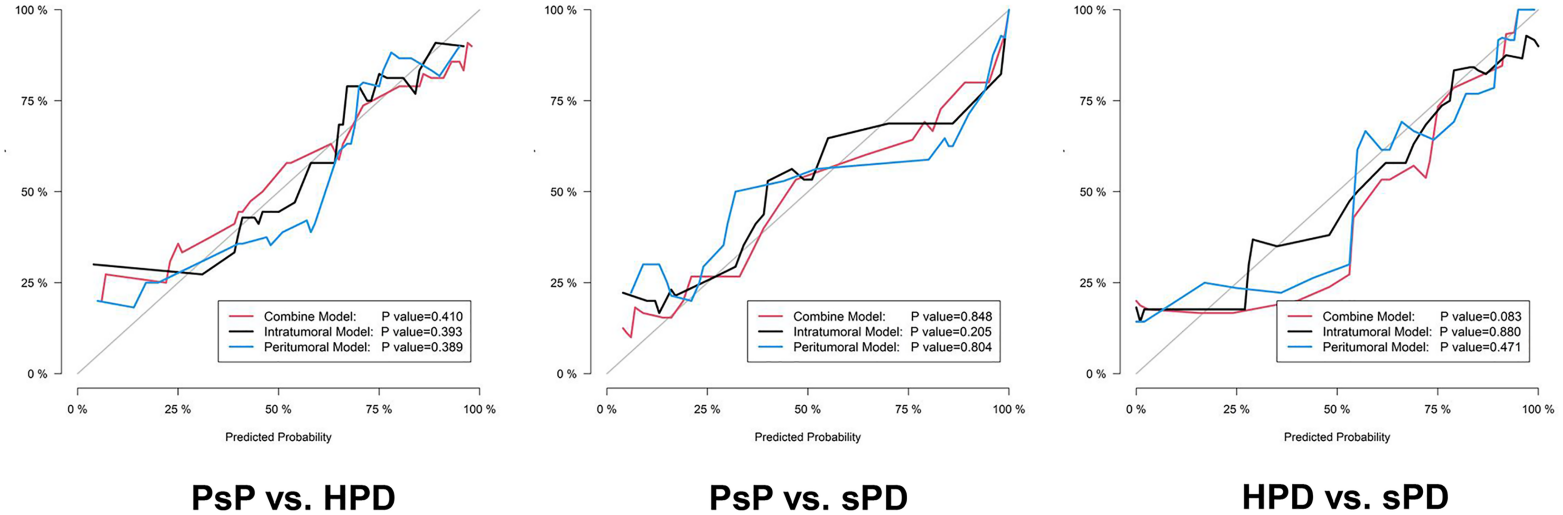
The calibration curves of the proposed radiomics models in the testing datasets. The 45° gray line indicates an ideal prediction. The black, blue, and red lines represent the intratumoral, peritumoral, and combined model predicted results, respectively. The X axis represents the predicted probability, and the Y axis represents true probability. The p value was derived from the Hosmer–Lemeshow test.

**Figure 6:**
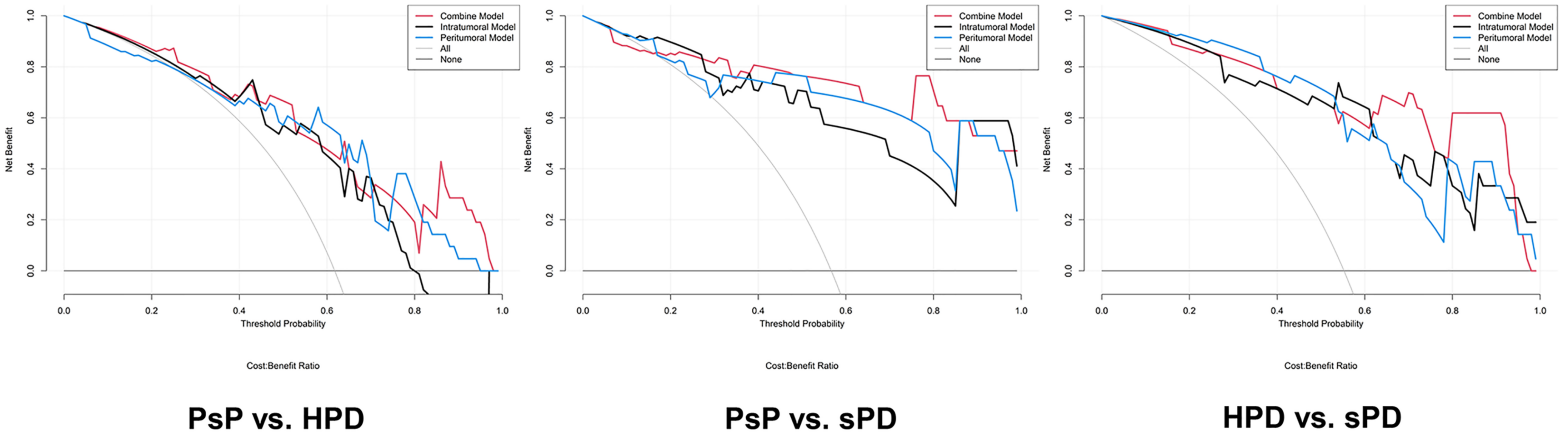
Decision curve analysis for the radiomics signatures in the testing datasets. The result of the decision curve analysis indicated that the prediction of PsP and HPD using the combined RS can give more net benefit than by treating none or all patients in both training and testing datasets.

The distribution of the combined RSs in the training and testing datasets is shown in **Figure S2**. The RSs derived from intra-tumoral, peri-tumoral, and combined models were all significantly higher in patients with HPD than those with PsP in both training and testing datasets (all p < 0.017 after Bonferroni correction). According to the maximum Youden’s index, 0.109, 0.386, and −0.298 were respectively set as the optimal cutoff values in the intra-, peri-tumoral, and combined models (**Tables 3-1**). **Table 4**, **Figure S3** and **Figure 4** show the discriminative performance of the models. The combined RS extracted from the intra- and peri-tumoral models yielded the highest AUC of 0.834 and an ACC of 0.827 in the training datasets, which were higher than those of the intra-tumoral model (yielded an AUC value of 0.804 and an ACC value of 0.733) alone. However, the RS extracted from the peri-tumoral model yielded the highest AUC of 0.848, along with a lower ACC of 0.773 than those of combined RS. Considering AUC and ACC together, a corresponding result was also found in testing datasets. The combined RS yielded the highest AUC value of 0.835 and an ACC value of 0.794, which were higher than those of intra- (yielded an AUC of 0.769 and ACC of 0.735) and peri-tumoral (yielded an AUC of 0.824 and ACC of 0.765) models alone. Interestingly, the RS extracted from the peri-tumoral model yielded a higher AUC than that of the intra-tumoral model in both training and testing datasets. The calibration curve indicated the good fitness of the conventional model with a p value of the Hosmer–Lemeshow test bigger than 0.05, which is shown in **Figure S4** and **Figure 5**. The clinical usefulness is shown as a decision curve in **Figure S5** and **Figure 6**.

**Table 3-1:** The distribution of the radiomics scores for PsP vs HPD in the training and testing datasets.

RS	Cutoff	Training dataset (N=75)			Testing dataset (N=34)			P value
		PsP	HPD	P value	PsP	HPD	P value	
**Intra-tumoral**	0.109	-0.56 (-0.78, 0.44)	1.19 (0.13, 2.67)	<0.001^*^	0.00 (-0.66, 1.08)	1.20 (0.92, 2.04)	0.009^*^	0.309
**Peri-tumoral**	0.386	-0.61 (-1.56, 0.43)	1.24 (0.41, 2.52)	<0.001^*^	-1.66 (-3.80, 0.13)	0.82 (0.07, 2.48)	0.002^*^	0.278
**Combined**	-0.298	-0.53 (-0.94, 0.41)	1.34 (0.18, 2.43)	<0.001^*^	-0.38±0.96	0.88±0.94	0.001^*^	0.243

#### RS for Discriminating PsP From sPD

Similar results were observed in PsP *vs*. sPD. As shown in **Table 4**, **Figure S3** and **Figure 4**, the combined RS yielded the highest AUC of 0.923 and an ACC of 0.868, which were higher than those of the intra- (yielded an AUC of 0.902 and an ACC of 0.838) and peri-tumoral (yielded an AUC of 0.912 and an ACC of 0.868) models alone. The peri-tumoral RS yielded a higher AUC value than that of the intra-tumoral model in both training and testing datasets. The RSs derived from the combined region were all significantly higher in patients with sPD than those with PsP in both training and testing datasets (**Figure S2**). The optimal cutoff values in the intra-, peri-tumoral, and combined model are displayed in **Table 3-2**. The calibration curve and decision curve are shown in **Figures S4**, **S5** and **Figures 5**, **6**.

**Table 3-2:** The distribution of the radiomics scores for PsP vs HPD in the training and testing datasets.

RS	Cutoff	Training dataset (N=68)			Testing dataset (N=30)			P value
		PsP	sPD	P value	PsP	sPD	P value	
**Intra-tumoral**	-0.376	-1.17 (-1.89, -0.46)	2.49 (0.16, 6.15)	<0.001^*^	-0.69 (-2.27, -0.36)	5.68 (0.26, 7.55)	<0.001^*^	0.723
**Peri-tumoral**	0.418	-1.36 (-1.91, 0.02)	3.43 (0.68, 7.28)	<0.001^*^	-0.49 (-1.45, 0.16)	5.65 (2.87, 8.47)	<0.001^*^	0.203
**Combined**	0.541	-1.43 (-2.37, -0.78)	2.56 (0.95, 11.66)	<0.001^*^	-1.76 (-3.03, -0.59)	3.95 (1.61, 11.87)	<0.001^*^	0.877	

#### RS for Discriminating HPD From sPD

The RSs derived from the combined region were all significantly higher in patients with sPD than those with HPD in both training and testing datasets (**Figure S2**). According to the maximum Youden’s index, 0.741, -0.154, and 1.325 were respectively set as the optimal cutoff values in the intra-, peri-tumoral, and combined models (**Table 3-3**). The combined RS yielded the highest AUC of 0.959 and an ACC of 0.894, which were higher than those of intra- (yielded an AUC of 0.911 and ACC of 0.824) and peri-tumoral (yielded an AUC of 0.894 and ACC of 0.835) models alone. The intra-tumoral RS yielded a higher AUC value than that of peri-tumoral RS in training datasets but lower in testing datasets (**Table 4**, **Figure S3** and **Figure 4**).

**Table 3-3:** The distribution of the radiomics scores for HPD vs sPD in the training and testing datasets.

RS	Cutoff	Training dataset (N=85)			Testing dataset (N=38)			P value
		HPD	sPD	P value	HPD	sPD	P value	
**Intra-tumoral**	0.741	-2.10±2.64	2.20±2.38	<0.001^*^	-1.25 (-5.95, 0.06)	1.86 (0.63, 3.87)	<0.001^*^	0.965
**Peri-tumoral**	-0.154	-1.01 (-15.76, -0.25)	1.37 (0.53, 3.38)	<0.001^*^	-6.44 (-29.39, 0.00)	1.92 (0.41, 3.28)	<0.001^*^	0.878
**Combined**	1.325	-2.84 (-21.49, -0.80)	3.52 (1.75, 5.01)	<0.001^*^	-18.12 (-26.04, 0.53)	8.14 (3.03, 9.66)	<0.001^*^	0.345

RS, radiomics signature; PsP, pseudoprogression; HPD, hyperprogression disease; sPD, standard progression disease; * indicated significant differences; Results for normal and non-normal distributions are means ± standard deviation and quartiles

**Table 4:** The discriminative performance of the models in the training and testing datasets.

Radiomics signatures		Training datasets						Testing datasets					
		AUC (95% CI)	ACC	SEN	SPE	PPV	NPV	AUC (95% CI)	ACC	SEN	SPE	PPV	NPV
**PsP *VS.* HPD**	**Intra-tumoral**	0.804 (0.717, 0.881)	0.733	0.739	0.724	0.810	0.636	0.769 (0.602, 0.913)	0.735	0.857	0.538	0.750	0.700
	**Peri-tumoral**	0.848 (0.770, 0.918)	0.773	0.783	0.759	0.837	0.688	0.824 (0.688, 0.941)	0.765	0.714	0.846	0.882	0.647
	**Combined**	0.834 (0.746, 0.914)	0.827	0.935	0.655	0.811	0.864	0.835 (0.704, 0.942)	0.794	0.905	0.615	0.792	0.800
**PsP *VS.* sPD**	**Intra-tumoral**	0.902 (0.837, 0.957)	0.838	0.872	0.793	0.850	0.821	0.891 (0.788, 0.973)	0.833	0.882	0.769	0.875	0.786
	**Peri-tumoral**	0.912 (0.846, 0.966)	0.868	0.769	1.000	1.000	0.763	0.900 (0.794, 0.981)	0.833	0.824	0.846	0.833	0.833
	**Combined**	0.923 (0.865, 0.972)	0.868	0.821	0.931	0.941	0.794	0.919 (0.813, 0.991)	0.867	0.824	0.923	0.933	0.800
**HPD *VS.* sPD**	**Intra-tumoral**	0.911 (0.857, 0.954)	0.824	0.717	0.949	0.943	0.740	0.891 (0.797, 0.966)	0.763	0.714	0.824	0.833	0.750
	**Peri-tumoral**	0.894 (0.833, 0.945)	0.835	0.870	0.795	0.833	0.838	0.899 (0.809, 0.969)	0.763	0.810	0.706	0.773	0.700
	**Combined**	0.959 (0.925, 0.986)	0.894	0.804	1.000	1.000	0.812	0.933 (0.863, 0.985)	0.842	0.857	0.824	0.857	0.824

Psp, pseudo-progression; HPD, hyper-progression disease; sPD, standard progression disease; ROC, receiver operating characteristic; AUC, area under ROC curve; ACC, accuracy; SPE, specificity; SEN, sensitivity; PPV, positive predictive value; NPV, negative predictive value.

## Discussion

In this multicenter study, we investigated the ability of pretreatment CT-based RSs extracted from intra- and peri-tumoral regions to predict atypical responses to ICI in multiple solid tumors. Our findings showed that the peri-tumoral regions have additional predictive values relative to the intra-tumoral regions in different immunotherapy responses, especially for PsP datasets.

As shown above, AUC values ranged from 0.834 to 0.959, whereas ACCs ranged from 0.827 to 0.894 in the combined RSs of the intra- and peri-tumoral regions outperformed than either of them alone. Besides, peri-tumoral RS showed a higher AUC (0.848 *vs*. 0.835) than combined RS in PsP *vs*. HPD, but lower ACC (0.773 *vs*. 0.827). In general, it is clear that the combination of intra- and peri-tumoral regions yielded the overall best classification performance. Noticeably, the most predictive RSs were found to be within an immediate distance of 5 mm from the lesion in predicting PsP when compared with either HPD or sPD, suggesting that features from the peri-tumoral region may have unique power in identifying PsP.

Radiomics is an approach involving a computerized extraction of certain quantitative imaging features, which has shown promise in predicting as well as monitoring treatment response (27). For predicting PsP, our results are in line with the study by Sun et al., who used radiomics from the peri-tumoral region to detect CD8 cells and predict immunotherapy response in multiple datasets (28). Similar results are available in research by Tunali et al.; the authors validated peri-tumoral features highly associated with the tumor-infiltrating lymphocyte (TIL) density on biopsy samples, which may provide a better understanding of the underlying biology (29). More specifically, the immediate surrounding tumor immune microenvironment (TIME) may offer unique information prior to administration of ICI that potentially decodes TILs. However, Shen and his colleagues constructed RS for predicting lymph node metastasis of patients with esophageal cancer before surgery from intra- and peri-tumoral areas and found that the former had a better performance in their research (30). In theory, due to the multiregional and microenvironmental heterogeneity in malignant tumors, it is reasonable to speculate that features from the whole tumor and peri-tumoral regions could have a comprehensive understanding of pathophysiology and the best performance in predicting response than those from only single regions (31). Although the biology of PsP and HPD is nevertheless to be understood, many of the present theories hypothesize that various immunoregulatory cells within the TIME could also be liable for this phenomenon (32). Interestingly, there is increasing evidence that TILs could be translated into certain quantitative features from the peri-tumoral region on CT-based radiomics, while further study is still needed (33).

PsP was first described on immunotherapy of the CTLA-4 inhibitor in melanoma, with a patient who experienced an enlargement of a cutaneous lesion after initial treatment, followed by a long-term stability (5). Afterward, PsP was used to describe as clinically improved or stable after a primary disease progression. The incidence of PsP cases observed in our study (8.25%) is consistent with the rate of this phenomenon observed in previous literatures as ranging from 1.1% to 9.1% across multiple solid tumor types (34). However, it should be noted that the incidence of PsP was underestimated in most clinical practices. For instance, PsP can imitate true progression radiographically and may be misclassified as a non-responder then excluded from immunotherapy by an inexperienced physician according to primitive WHO or RECIST criteria. Although iRECIST was proved superior to RECIST1.1 in identifying PsP, it requires an additional 4–8-week reassessment in cases of suspected progression causing an extra cost and a time leg on a potentially ineffective therapy (21). Our results indicate that radiomics can predict PsP who received ICI and may supplement conventional response evaluation criteria.

Actually, PsP is not a real tumor progression. The mechanism behind PsP could be that tumors could have ongoing growth until the activation of effective antitumor immune responses develops (35). Another explanation could be the infiltration of T cells into tumors, leading to a transient increase in tumor burden rather than true proliferation of tumor cells (36). The second hypothesis was later confirmed on tumor biopsies from patients with melanoma experiencing transient progression on a CTLA-4 inhibitor, showing an acute inflammatory reaction with lymphocyte infiltration.

Totally 9 of the 16 radiomics features were extracted from peri-tumoral regions in PsP *vs*. HPD and PsP *vs*. sPD groups that may be capturing data related to the TIME. Of note, the glszm_ZoneEntropy feature has been selected from both intra- and peri-tumoral regions in PsP *vs*. HPD, which was often appeared in tumor grading or staging and differentiation diagnosis (37, 38). Gldm_SmallDependenceEmphasis was used for predicting the genetic mutation status in NSCLC patients (39). Moreover, first-order and shape features quantify the range of gray values in the ROI which reflect the degree of heterogeneity of the tumor (40, 41). In a few clinical trials, PsP has been reported to be more common in younger patients, which may be due to the better reactivity of the immune system and may occur at any time after the start of treatment. However, there was no significant difference in our study, probably due to the limited sample size (42).

Another pattern of atypical responses called HPD was first reported in a case study of NSCLC on nivolumab treatment, with the observation that the patient seems to have an accelerated tumor growth rate after the initiation of ICI (43). The incidence of HPD from 4% to 29% of patients in various cancer types on ICI has been reported in several studies, which is higher than that of PsP (44). Unlike PsP followed subsequently by tumor regression, HPD represents true tumor growth and deserves more attention because patients experiencing HPD have a significantly shorter OS than sPD (3.6 *vs*. 6.2 months), suggesting that HPD has a deleterious effect and that it should be treated as a therapeutic emergency (45). Several mechanisms of HPD such as T cell exhaustion and expansion, aberrant inflammation and oncogenic pathway activation, and modulation of pro-tumorigenic immune subdatasets have been proposed (44). However, some previous reports suggested that being female, advanced age (>65 years), monotherapy, and epidermal growth factor receptor (EGFR) alterations were associated with HPD. However, the reports from different studies sometimes oppose each other (44, 46). We could not find any relationship between clinical variables and HPD in our study, probably related to the definition of HPD and the heterogeneity of the population enrolled. In contrast to predict PsP, the intra-tumoral RS have a similar performance with peri-tumoral RS, suggesting that the difference in heterogeneity plays a leading role between HPD and sPD, which could be explained by glszm_SizeZoneNonUniformityNormalized and coarseness features (both have higher values indicating more heterogeneity in the imaging). Recently, Tunali et al. reviewed pretreatment contrast-enhanced CT scans and radiomics features of 228 NSCLC patients, and they used parameters derived from both the tumor and tumor border regions to distinguish five HPD patients from non-HPD with an AUC of 0.865 (47). However, it was not capable to be applied because of shortages in cases and model construction.

Nowadays, some approaches are exploratorily used to identify atypical responses to immunotherapy. The first method is liquid biopsies, such as circulating tumor DNA (ctDNA), which was reported to decrease greatly in nine PsP patients with melanoma receiving ICI (47). However, different tumor types or inhibitors are required to further validate the relationship between ctDNA and PsP under immunotherapy. The second method is response evaluation criteria for immunotherapy, such as iRECIST (21). These criteria allow iUPD patients to continue treatment and reevaluate their responses with a time lag (after 4–8 weeks) or unneeded immune-related adverse effects (irAE). The third method is tumor genomic biomarkers, such as MDM2/MDM4 amplifications and EGFR alterations (48). Kato et al. performed next-generation sequencing of four patients with HPD revealed MDM2/MDM4 amplifications in 2 patients and EGFR amplification in one patient. In contrast, Kim et al. found no MDM2/MDM42 amplifications in the 18 patients with HPD (49). Unlike the above noninvasive methods, biopsies of tumors from some patients suspected of PsP have been found to contain dense inflammatory infiltrates or necrosis, instead of increased malignant load. Despite this, biopsies are often limited by a relatively small tissue sample and spatial heterogeneity and carry a procedure risk.

PsP and HPD are both atypical responses to immunotherapy, and oncologists should be conscientious to not confuse them with sPD, so as to avoid changing treatment too early for PsP, or too late in case of (hyper-)progressive disease. To this end, we aimed to use CT imaging, since it is routinely, noninvasive, and informative of the entire tumor burden and can be performed serially. To our knowledge, this is the first study to explore the ability of RS for prediction of atypical responses to ICI. Our findings potentially hold significant clinical applications, because they could provide a clinical framework for the pretreatment identification of atypical responses.

There are several limitations in our study. First is the retrospective nature and relatively small number of atypical responses, which limited the ability to perform stratified analyses, such as unitary tumor. However, we aim to test the true generalization performance of the classifier across multitumor interspecifics. Second, we only explored the radiomics features from pretreatment CT imaging; perhaps different or more information would be obtained with CT evaluations after ICI, although as claimed by the principle of “first, do no harm.” Third, lack of LDH, PD-L1 expression, and tumor mutation burden (TMB) in most patients in these retrospective datasets limit the assessment of prediction values. Fourth, the peri-tumoral features were only extracted within an immediate distance of 5 mm from the lesion. It is unclear whether other distances perform a superior prediction ability. Overall, validation of these radiomics biomarkers still needs to be done on larger multisite datasets.

## Conclusion

In conclusion, atypical responses to ICI are not uncommon phenomena observed with the incidence of 8.25% in PsP and 10.44% in HPD. The present preliminary study suggested that pretreatment CT-based radiomics provided a potential tool to differentiate among PsP, HPD, and sPD, thereby providing possibility for the prediction of atypical responses to ICI. In addition, RS derived from the peri-tumoral outperformed intra-tumoral region in identifying PsP, and the combined RS outperformed those from either intra- or peri-tumoral alone which may provide a more comprehensive characterization of atypical responses to ICI.

## Data Availability Statement

The original contributions presented in the study are included in the article/**Supplementary Material**. Further inquiries can be directed to the corresponding authors.

## Ethics Statement

This retrospective study was accepted by our institutional review committee (LNCH-20200611), and the prerequisite for obtaining informed consent was waived. The ethics committee waived the requirement of written informed consent for participation.

## Author Contributions

SH, TY, and YHL contributed to the conception and design of the study. All authors organized the database. LHW, SH, and YG performed the statistical analysis. YF and QL wrote the first draft of the manuscript. SH, TY, and YHL reviewed the manuscript. All authors contributed to the article and approved the submitted version. SH, YF, and QL have contributed equally to this work. All authors contributed to the article and approved the submitted version.

## Funding

This study was supported by the Wu Jieping Medical Foundation (No. 320.6750.2020-08-24).

## Conflict of Interest

Author YG was employed by company GE Healthcare.

The remaining authors declare that the research was conducted in the absence of any commercial or financial relationships that could be construed as a potential conflict of interest.

## Publisher’s Note

All claims expressed in this article are solely those of the authors and do not necessarily represent those of their affiliated organizations, or those of the publisher, the editors and the reviewers. Any product that may be evaluated in this article, or claim that may be made by its manufacturer, is not guaranteed or endorsed by the publisher.
