# Supplementary material for: CT-Based Peritumoral and Intratumoral Radiomics as Pretreatment Predictors of Atypical Responses to Immune Checkpoint Inhibitor Across Tumor Types: A Preliminary Multicenter Study
Source: Front Oncol. 2021 Oct 18;11:729371. doi: 10.3389/fonc.2021.729371 (PMC8560023; doi:10.3389/fonc.2021.729371)
Supplement: Supplementary file 1 [file DataSheet_1.docx]

Supplementary Material

# Supplementary Data

# CT scanning protocol

- 120 kVp with tube current adjusted automatically
- Reconstruction thickness: 5mm for Discovery CT750 scanner and Canon Aquilion ONE scanner
- Field of view (FOV): 36.5 cm to 44 cm
- Matrix: 512×512
- Reconstruction Kernel: Standard Algorithm (Std for GE, B30f for Siemens, or other equivalent)

**Supplementary Formula**

**Formula S1**

**The calculation formula of RSs for discriminating PsP from HPD were as follows:**

**Intra-tumoral RS (PsP vs HPD)** = 0.748 -

0.569 * original_glszm_ZoneEntropy -

0.356 * original_glszm_SizeZoneNonUniformity +

0.740 * original_shape_SurfaceVolumeRatio +

0.829 * original_firstorder_90Percentile

**Peri-tumoral RS (PsP vs HPD)** = 0.683 -

0.941 * original_glszm_ZoneEntropy -

0.330 * original_firstorder_Skewness -

0.776 * original_shape_MeshVolume +

1.600 * original_firstorder_90Percentile

**Combined RS (PsP vs HPD)** = 0.701 -

0.484 * original_glcm_SumAverage +

0.700 * original_firstorder_Variance -

0.530 * original_firstorder_Range -

0.031 *original_gldm_SmallDependenceEmphasis +

0.694 *original_glszm_ZoneEntropy

**Formula S2**

**The calculation formula of RSs for discriminating PsP from sPD were as follows:**

**Intra-tumoral RS (PsP vs sPD)** = 1.370 -

1.530 * original_glcm_Imc1 -

0.427 * original_glszm_GrayLevelNonUniformity +

1.524 * original_glcm_JointEnergy +

1.571 * original_gldm_LowGrayLevelEmphasis +

0.577 * original_glszm_SizeZoneNonUniformityNormalized +

0.873 * original_glcm_InverseVariance +

2.661 * original_firstorder_RootMeanSquared

**Peri-tumoral RS (PsP vs sPD)** = 1.876 -

0.507 * original_glszm_SizeZoneNonUniformityNormalized -

0.811 * original_gldm_DependenceNonUniformity +

1.153 * original_glrlm_ShortRunLowGrayLevelEmphasis +

2.013 * original_firstorder_RootMeanSquared +

3.319 * original_glcm_MaximumProbability -

0.170 * original_shape_SurfaceVolumeRatio +

0.383 * original_firstorder_Maximum +

0.081 * original_gldm_DependenceVariance

**Combined RS (PsP vs sPD)** = 2.274 -

1.471 * original_firstorder_Skewness -

0.030 * original_glrlm_LongRunHighGrayLevelEmphasis -

3.600 * original_gldm_HighGrayLevelEmphasis -

2.456 * original_gldm_DependenceVariance +

0.284 * original_glszm_LargeAreaLowGrayLevelEmphasis -

2.636 * original_shape_Sphericity +

0.012 * original_firstorder_Range -

1.025 * original_firstorder_Uniformity -

0.121 * original_glrlm_LongRunEmphasis +

0.367 * original_gldm_SmallDependenceLowGrayLevelEmphasis -

0.166 * original_glszm_LargeAreaLowGrayLevelEmphasis

**Formula S3**

**The calculation formula of RSs for discriminating HPD from sPD were as follows:**

**Intra-tumoral RS (HPD vs sPD)** = 0.227 +

0.650 * original_glszm_SizeZoneNonUniformityNormalized +

0.536 * original_glszm_SmallAreaEmphasis +

0.167 * original_firstorder_RootMeanSquared -

1.405 * original_glcm_Correlation +

1.885 * original_firstorder_Minimum -

1.468 * original_glszm_LowGrayLevelZoneEmphasis +

1.059 * original_ngtdm_Coarseness

**Peri-tumoral RS (HPD vs sPD)** = - 1.681 +

4.026 * original_firstorder_Mean +

0.641 * original_glszm_SizeZoneNonUniformityNormalized +

1.286 * original_gldm_DependenceNonUniformityNormalized -

2.689 * original_gldm_LargeDependenceLowGrayLevelEmphasis +

1.834 * original_glcm_Imc1-

0.249 * original_glcm_MaximumProbability +

0.041 * original_shape_SurfaceVolumeRatio +

0.804 * original_firstorder_Maximum +

0.957 * original_ngtdm_Coarseness

**Combined RS (HPD vs sPD)** = - 1.8795 - 0.822 * original_firstorder_Mean +

0.355 * original_glrlm_ShortRunEmphasis -

2.215 * original_glrlm_RunVariance -

4.411 * original_glszm_GrayLevelNonUniformity +

6.377 * original_glcm_Imc1-

1.731 * original_glcm_JointAverage +

1.606 * original_glcm_ClusterProminence +

2.495 * original_firstorder_Uniformity -

2.289 * original_glrlm_RunLengthNonUniformityNormalized +

0.451 * original_glrlm_LongRunEmphasis +

0.908 * original_gldm_DependenceNonUniformity +

2.349 * original_glszm_GrayLevelNonUniformity

# Supplementary Figures


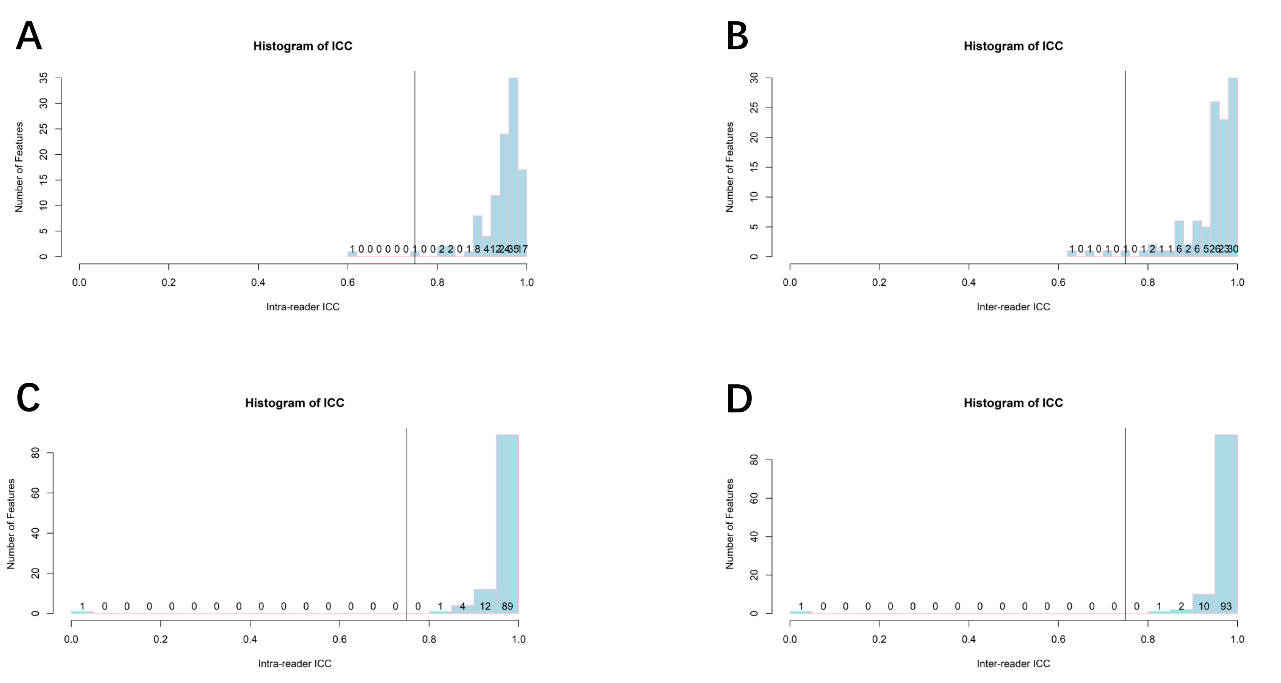


**Figure S1.** Histogram of Intra- and interclass correlation coefficients in intra-tumoral (A and B) and peri-tumoral (C and D) region.


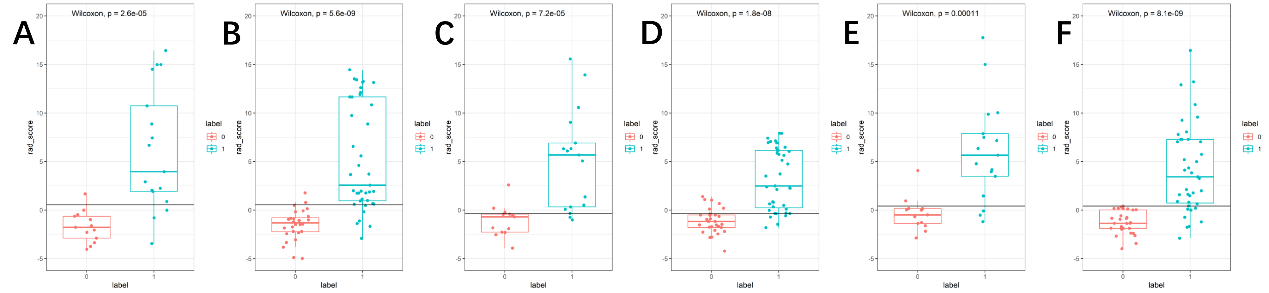


**Figure S2.** Box and whisker plots depict Radscore comparison between PsP, HPD and sPD. The distribution of combined RSs for discriminating Psp from HPD in the training (A) and testing (B) datasets. The distribution of combined RSs for discriminating Psp from sPD in the training (C) and testing (D) datasets. The distribution of combined RSs for discriminating HPD from sPD in the training (E) and testing (F) datasets.


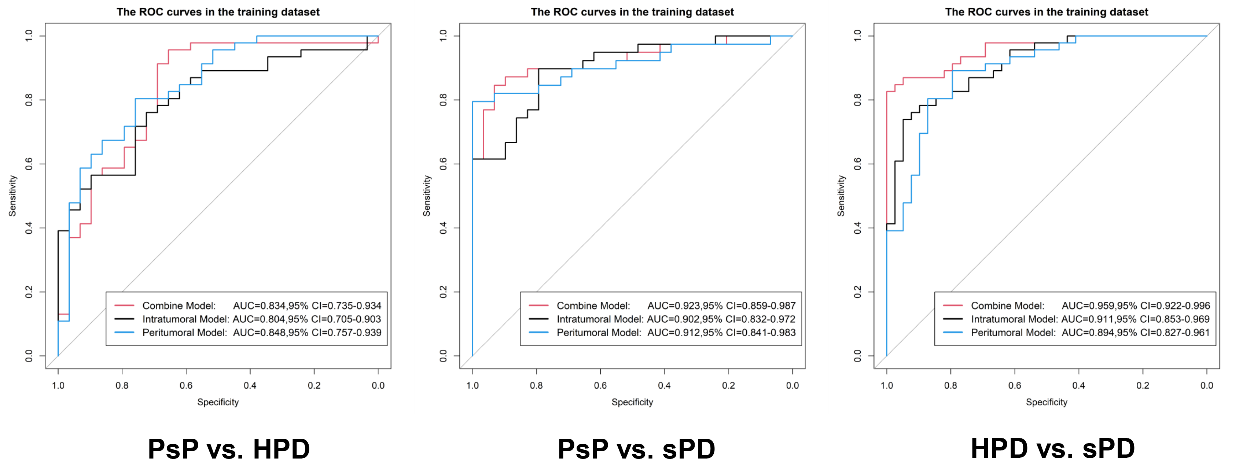


**Figure S3.** Receiver operating characteristic (ROC) curves of the radiomics signatures in the training dataset.


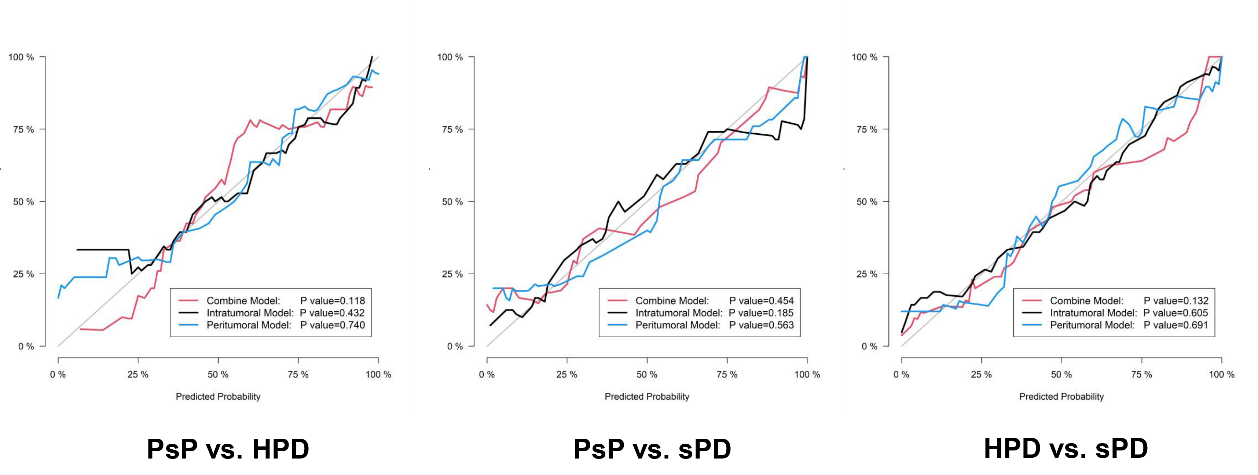


**Figure S4.** Calibration test of the radiomics signatures in the training dataset.

##
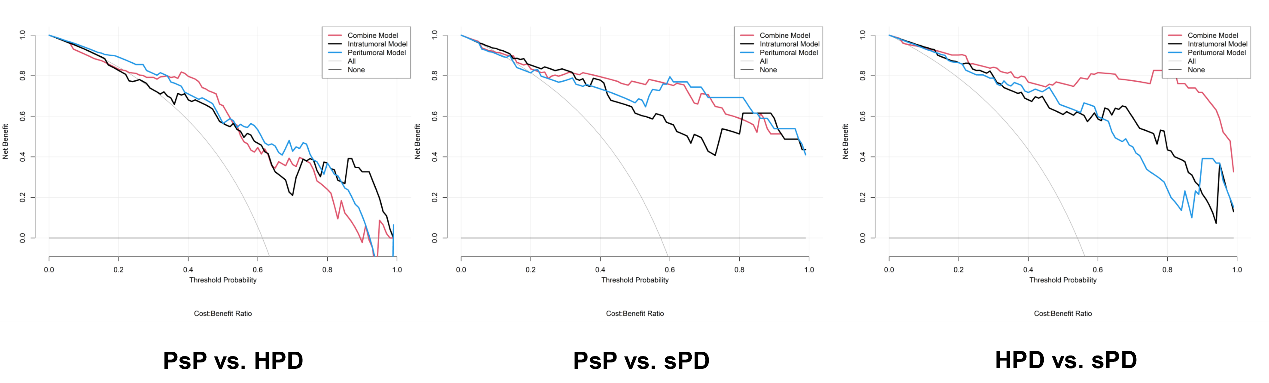


## Figure S5. Decision curves analysis for the radiomics signatures in the training dataset.

## Supplementary Table

**Table S1. Featrues characteristics of radiomics signatures in the training dataset**

| **Groups** | **Feature Names** | | |
| --- | --- | --- | --- |
|  | **Intra-tumoral** | **Peri-tumoral** | **Combined** |
| **PsP vs HPD** | **glszm_ZoneEntropy** | **glszm_ZoneEntropy** | **glcm_SumAverage** |
|  | **glszm_SizeZoneNonUniformity** | **firstorder_Skewness** | **firstorder_Variance** |
|  | **shape_SurfaceVolumeRatio** | **shape_MeshVolume** | **firstorder_Range** |
|  | **firstorder_90Percentile** | **firstorder_90Percentile** | **gldm_SmallDependenceEmphasis** |
|  |  |  | **glszm_ZoneEntropy** |
| **PsP vs sPD** | **glcm_Imc1** | **glszm_SizeZoneNonUniformityNormalized** | **firstorder_Skewness** |
|  | **glszm_GrayLevelNonUniformity** | **gldm_DependenceNonUniformity** | **glrlm_LongRunHighGrayLevelEmphasis** |
|  | **glcm_JointEnergy** | **firstorder_Maximum** | **gldm_HighGrayLevelEmphasis** |
|  | **gldm_LowGrayLevelEmphasis** | **firstorder_RootMeanSquared** | **gldm_DependenceVariance** |
|  | **glszm_SizeZoneNonUniformityNormalized** | **glcm_MaximumProbability** | **glszm_LargeAreaLowGrayLevelEmphasis** |
|  | **glcm_InverseVariance** | **shape_SurfaceVolumeRatio** | **shape_Sphericity** |
|  | **firstorder_RootMeanSquared** | **glrlm_ShortRunLowGrayLevelEmphasis** | **firstorder_Range** |
|  |  | **gldm_DependenceVariance** | **firstorder_Uniformity** |
|  |  |  | **glrlm_LongRunEmphasis** |
|  |  |  | **gldm_SmallDependenceLowGrayLevel Emphasis** |
|  |  |  | **glszm_LargeAreaLowGrayLevelEmphasis** |
|  |  |  | **glszm_GrayLevelNonUniformity** |
| **HPD vs sPD** | **glszm_SizeZoneNonUniformityNormalized** | **firstorder_Mean** | **firstorder_Mean** |
|  | **glszm_SmallAreaEmphasis** | **glszm_SizeZoneNonUniformityNormalized** | **glrlm_ShortRunEmphasis** |
|  | **firstorder_RootMeanSquared** | **gldm_DependenceNonUniformityNormalized** | **glrlm_RunVariance** |
|  | **glcm_Correlation** | **gldm_LargeDependenceLowGrayLevelEmphasis** | **glszm_GrayLevelNonUniformity** |
|  | **firstorder_Minimum** | **glcm_Imc1** | **glcm_Imc1** |
|  | **glszm_LowGrayLevelZoneEmphasis** | **glcm_MaximumProbability** | **glcm_JointAverage** |
|  | **ngtdm_Coarseness** | **shape_SurfaceVolumeRatio** | **glcm_ClusterProminence** |
|  |  | **firstorder_Maximum** | **firstorder_Uniformity** |
|  |  | **ngtdm_Coarseness** | **glrlm_RunLengthNonUniformityNormalized** |
|  |  |  | **glrlm_LongRunEmphasis** |
|  |  |  | **gldm_DependenceNonUniformity** |

PsP, pseudoprogression; HPD, hyperprogression disease; sPD, standard progression disease; GLCM, Gray-Level Co-occurrence Matrix; GLRLM, Gray-Level Run-Length Matrix; GLSZM, Gray-Level Size Zone Matrix; NGTDM, Neighborhood Gray Tone Difference Matrix
